# Operational Constraints in Quantum Otto Engines: Energy-Gap Modulation and Majorization

**DOI:** 10.3390/e27060625

**Published:** 2025-06-12

**Authors:** Sachin Sonkar, Ramandeep S. Johal

**Affiliations:** Department of Physical Sciences, Indian Institute of Science Education and Research Mohali, Sector 81, Sahibzada Ajit Singh Nagar P.O. Box 140306, Punjab, India; ph19077@iisermohali.ac.in

**Keywords:** quantum Otto engine, majorization, swap engine

## Abstract

The performance of a quantum Otto engine is analyzed with regard to the constraints on the modulation of energy gaps relative to the changes in probability distributions at the two given heat reservoirs. We performed a detailed analysis with a generic three-level system (3LS), which serves as a non-trivial working medium with two energy gaps. A three-level Otto engine becomes feasible if at least one energy gap shrinks during the first quantum adiabatic stage. The operating regimes are derived for each allowed energy gap modulation, and majorization is observed to play a crucial role in determining the engine operation. This results in an enhanced Otto efficiency when the probability distributions fulfill the majorization condition. Finally, we show that our formalism applies to a swap engine based on a working medium composed of two 3LSs.

## 1. Introduction

A quantum system in thermal contact with two heat reservoirs was the first prototype [1] for a quantum heat engine, consistent with the second law of thermodynamics. Following this discovery and subsequent developments [2,3,4,5,6,7,8], the field of quantum thermodynamics has literally picked up steam and has seen rapid advances in recent years [9,10,11,12,13]. Quantum thermal machines explore the resourcefulness of quantum features, such as coherence and entanglement [5,14,15,16,17,18,19,20,21], for energy conversion purposes. Such machines are now being experimentally realized across various platforms [22,23,24,25,26,27,28,29,30,31,32,33,34,35,36,37,38,39,40]. The performance analysis of quantum heat engines, refrigerators, and heat pumps has thus emerged as a major sub-discipline in this domain, where the quantum Otto cycle has been widely studied using a variety of working media and reservoir configurations, both in the quasi-static as well as finite-time regimes [41,42,43,44,45,46,47,48,49,50,51,52,53,54,55,56,57,58,59,60,61,62,63,64,65,66,67,68,69,70,71]. In the paradigmatic model of a two-level Otto engine [54,64,72,73,74,75,76,77,78], net heat flow from a hot to a cold reservoir is ensured if the equilibrium ground state probability at the cold reservoir is higher than the corresponding probability at the hot reservoir. Specific working conditions for multi-level systems [19,41,44,45,53,57,79,80] are usually difficult to pin down, except when the energy spectrum changes in a specific way. Some many-body systems may yield to analytic treatments, see, e.g., refs. [55,81,82,83].

In particular, the technique of majorization [84,85,86,87,88,89,90,91,92] has been found to be useful in certain coupled-spins models [44,45]. The connection between the performance of a quantum heat engine and majorization was noted in ref. [44]. Subsequently, the concept of majorization was employed to characterize the performance of a spin-based quantum Otto engine (QOE) in ref. [45]. The majorization criterion has important implications for state transformation in various resource theories [93,94,95,96,97,98,99,100,101,102,103,104,105]. Additionally, majorization serves as a sufficient criterion for ordering the maximum work extraction from a finite quantum system when it is in an equal-energetic state [6,106]. This concept also proves valuable in deriving bounds on the ergotropy gap, with implications for witnessing and quantifying entanglement [107,108,109] and applications in models of quantum networks [110] and quantum batteries [111].

In this paper, we investigate the working conditions on a multilevel quantum system from the perspective of modulating the energy gaps. In this context, we address the following question: Given two heat reservoirs at different temperatures, what changes in the gaps are compatible with specific changes in the occupation probabilities? In our simplified semi-classical picture, the following assumptions are made. First, the interaction between the quantum system and the heat reservoirs is considered weak so that the Hamiltonian does not contain any such interaction term. Second, the energy gaps are modulated by an external field such that the level populations in the system are preserved. The average energy thus exchanged between the field and the system is interpreted as work only. We also neglect any energy cost for operating the external field. Since our system undergoes complete thermalization and there are no coherences generated in adiabatic steps, we are able to exclude any effects on performance due to coherence.

In other words, the state of a quantum system is described in terms of energy levels/gaps along with the relevant probability distribution. Since an Otto cycle constitutes processes that can be regarded as an exchange of only work or heat, the changes involving the gaps or the occupation probabilities represent two distinct types of controls in a quantum Otto cycle. We ascertain the permissible changes in the gaps vis-à-vis the difference between the hot and cold probability distributions. Our results show that starting from the initial state of the *n*-level system in equilibrium with the hot reservoir, at least one of the energy gaps must shrink in order to realize an Otto engine; the engine is not feasible if all the gaps expand during the first quantum adiabatic stage. Further, a definite majorization relation between the hot and cold distributions determines the feasibility of the engine. For an *n*-level system with non-increasing gaps during the first adiabatic stroke, majorization serves as a sufficient criterion. A detailed analysis of a three-level system reveals that when both gaps shrink, majorization remains a sufficient condition for the engine. However, when only one gap shrinks, it becomes a necessary condition. In the special case where one of the two gaps is fixed, majorization provides both a necessary and sufficient condition for the Otto engine.

The paper is organized as follows: Section 2 studies the performance of a QOE with an *n*-level as a working medium. In Section 3, the compatibility conditions between probability distributions at the two reservoirs and energy gaps for the feasibility of a three-level system (3LS) Otto engine are derived. In Section 4, we discuss bounds on the efficiency of the Otto engine and compare various configurations to identify optimal performance. It is observed that configurations yielding better performance are those where probabilities are distributed according to the majorization relation between hot and cold distributions. Section 5 discusses the mapping of QOE to a swap engine based on two 3LSs. The conclusions of our paper are presented in Section 6.

## 2. Quantum Otto Engine with n-Level System

The notions of heat and work play a central role in thermodynamics. Extracting heat from a hot reservoir ($Q_{1}>0$), a heat engine delivers a part of this energy as work (W<0) and dumps the unused heat into the cold reservoir ($Q_{2}<0$). So, energy conservation in the heat engine implies: $Q_{1}+Q_{2}+W=0$. In the quasi-static Otto cycle, each step is slow enough, and the notion of rates does not enter the formalism. Each of the compression/expansion stages involves a quantum adiabatic process, where an externally controllable parameter of the Hamiltonian can be varied. The quantum adiabatic theorem [112] ensures that this process does not generate transitions between energy levels, thus preserving their occupation probabilities. The remaining two steps in the cycle are the isochoric heating and cooling processes, where the system thermalizes with the hot and cold reservoirs, respectively.

**Figure 1:**
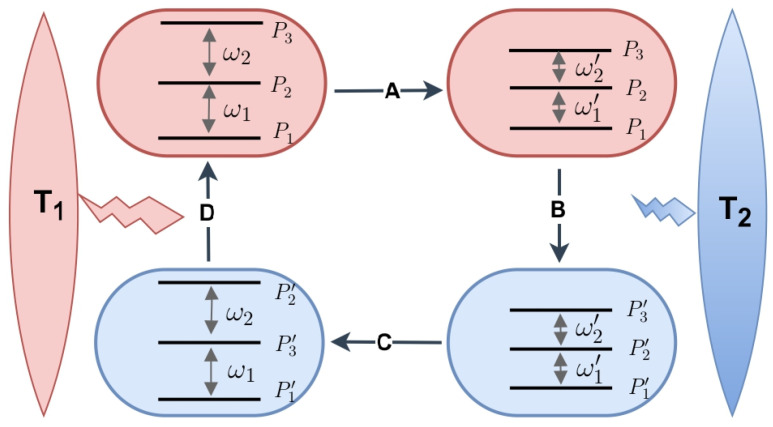
The schematic of a quantum Otto engine (QOE) based on a 3LS. Stages A and C are the first and the second quantum adiabatic processes, respectively, where the energy gaps interchange between values $\omega_{1}↔\omega_{1}^{\prime }$ and $\omega_{2}↔\omega_{2}^{\prime }$. Heat exchange at the cold ($T_{2}$) and the hot ($T_{1}$) reservoir occurs in stage B and D, respectively.

Let us consider an *n*-level quantum system with the Hamiltonian $H=\sum_{k}\varepsilon_{k}\left|k\rangle \langle k\right|$. Initially, the system is in a thermal state, $\rho =\sum_{k}P_{k}\left|k\rangle \langle k\right|$, corresponding to the hot temperature $T_{1}$. The occupation probability for an energy level with energy $\varepsilon_{k}$, is given as: $P_{k}=e^{-\varepsilon_{k}/T_{1}}/\sum_{k}e^{-\varepsilon_{k}/T_{1}}$ (in our system of units, Boltzmann’s constant is unity). The four stages of the Otto cycle are described as follows. In stage A, the system is disconnected from the hot reservoir and undergoes a quantum adiabatic process in which the energy gaps are modulated by some external fields so the energy eigenvalues become $\varepsilon_{k}^{\prime }$. In stage B, the system is brought into thermal contact with the cold reservoir at temperature $T_{2}\left(<T_{1}\right)$ until thermal equilibrium is achieved. In this stage, the energy eigenvalues remain at $\varepsilon_{k}^{\prime }$, while the occupation probabilities change from $P_{k}$ to $P_{k}^{\prime }=e^{-\varepsilon_{k}^{\prime }/T_{2}}/\sum_{k}e^{-\varepsilon_{k}^{\prime }/T_{2}}$. In stage C, the system is disengaged from the cold reservoir, and the energy levels are modulated back to their initial values $\varepsilon_{k}$ while the occupation probabilities $P_{k}^{\prime }$ remain unchanged. Finally, in stage D, the system is equilibrated with the hot reservoir, restoring the initial state ρ and completing the heat cycle (a schematic of the cycle given in Figure 1).

The general form of the heat and work contributions is based on the first law of thermodynamics [9]. For an *n*-level system, the heat exchanges during stages D and B, given by the difference of the final and initial mean energies of the system in that process, are

(1) $$\begin{array}{cc} Q_{1} & =\sum_{k=1}^{n}\varepsilon_{k}\left(P_{k}-P_{k}^{\prime }\right),\;\;\;\;\;Q_{2}=\sum_{k=1}^{n}\varepsilon_{k}^{\prime }\left(P_{k}^{\prime }-P_{k}\right). \end{array}$$

The net work extracted from the engine per cycle ($W\equiv -W=Q_{1}+Q_{2}$) is given by:

(2) $$W=\sum_{k=1}^{n}\left(\varepsilon_{k}-\varepsilon_{k}^{\prime }\right)\left(P_{k}-P_{k}^{\prime }\right).$$

W>0 is called the positive work condition (PWC) of the engine, along with $Q_{1}>0$ and $Q_{2}<0$. The efficiency of the engine is defined as $\eta =W/Q_{1}=1+Q_{2}/Q_{1}$.

To facilitate our analysis through the lens of majorization, we rewrite the above expressions as (derived in Appendix A):

(3) $$\begin{array}{cc} Q_{1} & =\sum_{j=1}^{n-1}\omega_{j}M_{j},\;\;Q_{2}=-\sum_{j=1}^{n-1}\omega_{j}^{\prime }M_{j} \end{array}$$

and

(4) $$\begin{array}{cc} W & =\sum_{j=1}^{n-1}\left(\omega_{j}-\omega_{j}^{\prime }\right)M_{j}, \end{array}$$

where $\omega_{j}=\varepsilon_{j+1}-\varepsilon_{j}$ and $\omega_{j}^{\prime }=\varepsilon_{j+1}^{\prime }-\varepsilon_{j}^{\prime }$ are the corresponding energy gaps of the 3LS at the hot and the cold reservoirs, respectively, and $M_{j}=\sum_{i=j+1}^{n}\left(P_{i}-P_{i}^{\prime }\right)$. We assume that the energy eigenvalues are given in increasing order: $\varepsilon_{1}<\varepsilon_{2}<\ldots <\varepsilon_{n}$, and $\varepsilon_{1}^{\prime }<\varepsilon_{2}^{\prime }<\ldots <\varepsilon_{n}^{\prime }$. Correspondingly, the occupation probabilities at the hot and cold reservoirs are ordered as $P_{1}>P_{2}>\ldots >P_{n}$, and $P_{1}^{\prime }>P_{2}^{\prime }>\ldots >P_{n}^{\prime }$, respectively. Then, the probability distribution *P* is said to be majorized (denoted as $P≺P^{\prime }$, as defined in Equation (A4)) by the probability distribution $P^{\prime }$, implying that $M_{j}\geq 0$ for j=1,2,…,n−1.

From Equation (3), we note that $P≺P^{\prime }$, implying $M_{j}\geq 0$, ensures that the heat exchange at the hot reservoir is always positive ($Q_{1}>0$), while at the cold reservoir, $Q_{2}<0$. However, it is evident from Equation (4) that the majorization relation itself does not guarantee positive work. For example, when all the gaps shrink $\left(\omega_{j}-\omega_{j}^{\prime }>0\right)$, or when some of the gaps shrink while the others are fixed $\left(\omega_{k}-\omega_{k}^{\prime }=0\right)$, the majorization relation ensures positive work extraction. Conversely, if any of the energy gaps increase in the first stage, the majorization condition does not guarantee positive work. Thus, we conclude that when the energy gaps during the first adiabatic stroke are non-increasing, majorization serves as a sufficient criterion for positive work extraction. In Appendix A, we solve the majorization relation for a general *n*-level system and derive a sufficient condition for positive work, expressed as $\omega_{j}^{\prime }\leq \omega_{j}\leq \left(T_{1}/T_{2}\right)\omega_{j}^{\prime }$, for j=1,2,…,n−1. In the special case where all the energy gaps change by the same ratio, majorization provides both the necessary and sufficient conditions for the engine operation. Determining the conditions on the engine parameters, such as the relation between reservoir temperatures and energy gaps, is a challenging task with a generic working medium. In the next section, we focus on the specific case of a three-level system as the working medium and show that majorization offers a solid framework for evaluating the relationships among these parameters.

## 3. Gap Modulation and Majorization in Three-Level System

A three-level system (3LS) has provided a convenient platform from which to examine the influence of population and coherence on the performance of a thermal machine [113,114,115,116,117,118,119], avoiding any contributions from quantum entanglement or quantum correlations between subsystems. A 3LS or a qutrit has important applications in quantum information processing [120,121,122]. It is proving to be a testbed for the study of heat transport and other nonequilibrium features in three heat reservoir set ups [123]. A 3LS may be modelled as a superconducting loop containing three Josephson junctions [124], or through a V-type level structure where the two degenerate upper levels are Raman-coupled via the lower level. More recently, ref. [125] reported the doubling of the power output of a continuous heat engine utilizing a degenerated V-type 3LS as compared to two independent two-level systems (see also [126]). We use a 3LS with two energy gaps as an illustrative example for analyzing the relation between energy-gap modulation and the probability distributions. Ref. [127] considered a quasi-static cycle allowing four possible changes in the energy gaps during the first adiabatic step of the Otto cycle. In the high temperature limit, the specific case of both gaps shrinking yields a positive work condition that can be looser than that of a two-level system. However, even for a 3LS, a complete characterization of the operating conditions of a quasi-static Otto cycle seems to be lacking in the literature (see ref. [128] for a relativistic generalization).

The Hamiltonian of a non-degenerate, three-level working medium is given by: $H=\sum_{k=1}^{3}\varepsilon_{k}\left|k\rangle \langle k\right|$. The energy eigenvalues and occupation probabilities are ordered as: $\varepsilon_{1}<\varepsilon_{2}<\varepsilon_{3}$ $\left(P_{1}>P_{2}>P_{3}\right)$ and $\varepsilon_{1}^{\prime }<\varepsilon_{2}^{\prime }<\varepsilon_{3}^{\prime }$ $\left(P_{1}^{\prime }>P_{2}^{\prime }>P_{3}^{\prime }\right)$. The lower and upper energy gaps are defined as: $\omega_{1}=\varepsilon_{2}-\varepsilon_{1}>0$ and $\omega_{2}=\varepsilon_{3}-\varepsilon_{2}>0$ (see Figure 1) with the sum, $\omega =\omega_{1}+\omega_{2}$. Similarly, $\omega_{1}^{\prime }=\varepsilon_{2}^{\prime }-\varepsilon_{1}^{\prime }>0$, $\omega_{2}^{\prime }=\varepsilon_{3}^{\prime }-\varepsilon_{2}^{\prime }>0$ and $\omega^{\prime }=\omega_{1}^{\prime }+\omega_{2}^{\prime }$. In terms of these gaps, the probability distributions can be written as: $P_{1}={\left(1+e^{-\omega_{1}/T_{1}}+e^{-\omega /T_{1}}\right)}^{-1},P_{3}={\left(1+e^{\omega_{2}/T_{1}}+e^{\omega /T_{1}}\right)}^{-1}$ and $P_{2}=1-P_{1}-P_{3}$. Likewise, $P_{1}^{\prime }={\left(1+e^{-\omega_{1}^{\prime }/T_{2}}+e^{-\omega^{\prime }/T_{2}}\right)}^{-1},P_{3}^{\prime }={\left(1+e^{\omega_{2}^{\prime }/T_{2}}+e^{\omega^{\prime }/T_{2}}\right)}^{-1}$ and $P_{2}^{\prime }=1-P_{1}^{\prime }-P_{3}^{\prime }$. Finally, the expressions for heat and work are given by

(5) $$\begin{array}{cc} Q_{1} & =\omega_{2}\left(P_{3}-P_{3}^{\prime }\right)+\omega_{1}\left(P_{1}^{\prime }-P_{1}\right), \end{array}$$

(6) $$\begin{array}{cc} Q_{2} & =-\omega_{2}^{\prime }\left(P_{3}-P_{3}^{\prime }\right)-\omega_{1}^{\prime }\left(P_{1}^{\prime }-P_{1}\right), \end{array}$$

(7) $$\begin{array}{cc} W\; & =\left(\omega_{2}-\omega_{2}^{\prime }\right)\left(P_{3}-P_{3}^{\prime }\right)+\left(\omega_{1}-\omega_{1}^{\prime }\right)\left(P_{1}^{\prime }-P_{1}\right). \end{array}$$

Note that the net work is the sum of the work performed in the adiabatic stages: $W=W_{1}+W_{2}$. However, in our analysis, we only require the total work to be extracted (W<0) and do not specify the sign taken by the individual work contributions. $W_{1}<0$ and $W_{2}>0$ are shown in Figure 1, only for concreteness.

### 3.1. Changing the Gaps Vis-à-Vis Probabilities

Assuming that both gaps undergo changes during the engine cycle, there are four possible ways (denoted as G-1,2,3,4) by which the gaps can change during the first adiabatic step:

G-1 Both gaps shrink: $\omega_{1}>\omega_{1}^{\prime },\;\omega_{2}>\omega_{2}^{\prime }$.

G-2 Lower gap shrinks, upper gap expands: $\omega_{1}>\omega_{1}^{\prime },\;\omega_{2}<\omega_{2}^{\prime }$.

G-3 Upper gap shrinks, lower gap expands: $\omega_{1}<\omega_{1}^{\prime },\;\omega_{2}>\omega_{2}^{\prime }$.

G-4 Both gaps expand: $\omega_{1}<\omega_{1}^{\prime },\;\omega_{2}<\omega_{2}^{\prime }$.

On the other hand, the changes in the occupation probabilities may belong to one of the following cases:

Case (a) $P_{1}>P_{1}^{\prime },\;\;P_{3}>P_{3}^{\prime },\;\;P_{2}<P_{2}^{\prime }$;

Case (b) $P_{1}<P_{1}^{\prime },\;\;P_{3}<P_{3}^{\prime },\;\;P_{2}>P_{2}^{\prime }$;

Case (c) $P_{1}<P_{1}^{\prime },\;\;P_{3}>P_{3}^{\prime }$;

Case (d) $P_{1}>P_{1}^{\prime },\;\;P_{3}<P_{3}^{\prime }$.

In Cases (a) and (b), a definite inequality between $P_{2}$ and $P_{2}^{\prime }$ holds due to the normalization condition on probabilities. On the other hand, for Cases (c) and (d), the inequality between $P_{2}$ and $P_{2}^{\prime }$ remains indefinite. Next, we ascertain the mutual compatibility between the conditions on gaps and the feasible cases for probabilities.

From the analysis of Case (a) (Appendix A.2), we deduce that for engine operation (W>0, $Q_{1}>0$ and $Q_{2}<0$), both energy gaps must shrink in the first adiabatic step. The constraint on the energy gap are given by $\omega_{1}>\omega_{1}^{\prime }$ and $\omega_{2}^{\prime }\left(T_{1}/T_{2}\right)>\omega_{2}>\omega_{2}^{\prime }$. The relation between occupation probability and energy gaps is derived as

(8) $$\frac{\omega_{2}^{\prime }}{\omega_{1}^{\prime }}>\frac{\omega_{2}}{\omega_{1}}>\frac{\omega_{2}-\omega_{2}^{\prime }}{\omega_{1}-\omega_{1}^{\prime }}>\frac{P_{1}-P_{1}^{\prime }}{P_{3}-P_{3}^{\prime }}.$$

Similarly, solving for Case (b) (Appendix A.3), we obtain $\omega_{2}>\omega_{2}^{\prime }$ and $\omega_{1}^{\prime }\left(T_{1}/T_{2}\right)>\omega_{1}>\omega_{1}^{\prime }$, and reveal that this scenario also requires both the gaps to shrink during the first adiabatic step. For this case, the relation between occupation probability and energy gaps is expressed as

(9) $$\frac{\omega_{1}^{\prime }}{\omega_{2}^{\prime }}>\frac{\omega_{1}}{\omega_{2}}>\frac{\omega_{1}-\omega_{1}^{\prime }}{\omega_{2}-\omega_{2}^{\prime }}>\frac{P_{3}^{\prime }-P_{3}}{P_{1}^{\prime }-P_{1}}.$$

For Case (c), which represents a majorization relation [84] ($P≺P^{\prime }$ as defined in Equation (A4)) between the hot and cold distributions of a 3LS. The said relation is usually expressed in terms of the following set of inequalities: $P_{3}\geq P_{3}^{\prime }$, $P_{2}+P_{3}\geq P_{2}^{\prime }+P_{3}^{\prime }$, and $P_{1}+P_{2}+P_{3}=P_{1}^{\prime }+P_{2}^{\prime }+P_{3}^{\prime }=1$. Clearly, these conditions also imply $P_{1}\leq P_{1}^{\prime }$. Thus, for Case (c), we obtain

(10) $$\frac{P_{3}}{P_{1}}>\frac{P_{3}^{\prime }}{P_{1}^{\prime }}\Rightarrow \frac{\omega }{T_{1}}<\frac{\omega^{\prime }}{T_{2}}.$$

From Equation (7), we see that majorization is a sufficient condition for PWC when both gaps shrink (G-1). Moreover, when only one gap shrinks while the other one expands (G-2 and G-3), PWC is a possible scenario. If the lower gap shrinks (G-2), then PWC requires that

(11) $$\frac{P_{1}^{\prime }-P_{1}}{P_{3}-P_{3}^{\prime }}>\frac{\omega_{2}^{\prime }-\omega_{2}}{\omega_{1}-\omega_{1}^{\prime }}.$$

On the other hand, when the upper gap shrinks (G-3), then PWC requires that

(12) $$\frac{P_{1}^{\prime }-P_{1}}{P_{3}-P_{3}^{\prime }}<\frac{\omega_{2}-\omega_{2}^{\prime }}{\omega_{1}^{\prime }-\omega_{1}}.$$

Consequently, when the hot and cold probabilities satisfy the majorization relation $P≺P^{\prime }$, then all three conditions (G-1,2,3) on the energy gaps are feasible. In this sense, the validity of a majorization relation gives us the maximal freedom to manipulate a 3LS for the purpose of the Otto engine.

**Figure 2:**
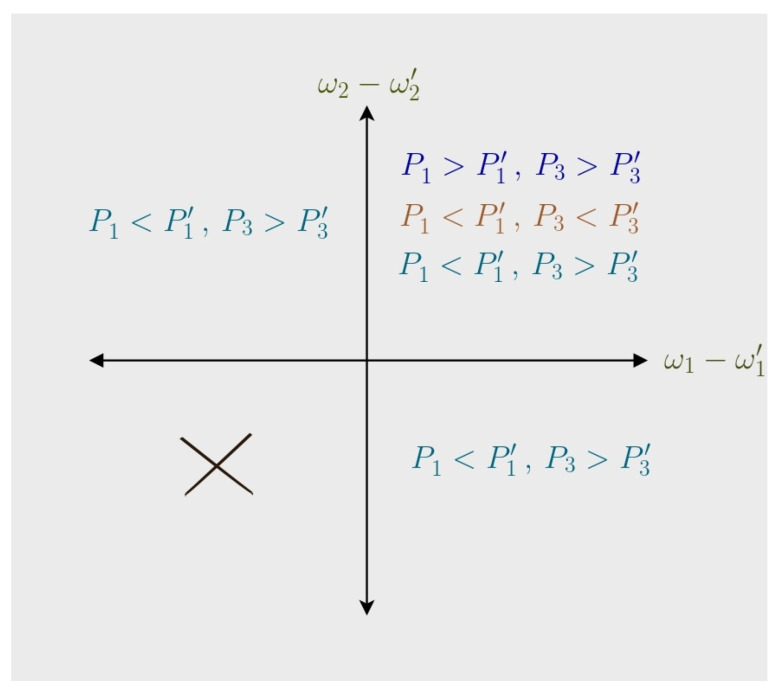
In the first quadrant, where both gaps shrink, Cases (a), (b), and (c) are all permissible. However, in the second and fourth quadrants, only the majorization relation ($P≺P^{\prime }$), i.e., Case (c), is allowed. In the third quadrant, where both gaps are expanding, work cannot be extracted.

Finally, we note that Case (d) results in $Q_{1}<0$ and $Q_{2}>0$, which makes it inconsistent with the operation of an engine. Also, for G-4, where both gaps expand, we find that the quantities $P_{3}>P_{3}^{\prime }$ and $P_{1}^{\prime }>P_{1}$ hold. Thus, Equation (7) results in work being negative, invalidating G-4 for the Otto engine. A graphical summary of the compatible changes in the gaps versus the changes in probabilities is given in Figure 2.

### 3.2. Case of a Fixed Gap

So far, in the above analysis, we have assumed that both gaps may undergo cyclic changes during the heat cycle. An interesting special case arises when one of the gaps stays fixed while the other shrinks. Note that the variable gap cannot expand if the machine is to work as an engine. Appendix A.4 shows that Cases (a) and (b) are not permissible when only one gap shrinks while the other stays fixed—only Case (c), satisfying the majorization condition, allows for positive work. Thus, majorization provides a necessary and sufficient condition for the engine when one gap is held fixed.

Finally, we consider the special case where the total gap is fixed, i.e., $\omega =\omega^{\prime }$. We then have one of the two conditions: either G-2 where $\omega_{1}>\omega_{1}^{\prime },\;\;\omega_{2}<\omega_{2}^{\prime }$, or G-3, for which $\omega_{1}<\omega_{1}^{\prime },\;\;\omega_{2}>\omega_{2}^{\prime }$. The expression for work, Equation (7), is now simplified to

(13) $$W=\left(\omega_{2}-\omega_{2}^{\prime }\right)\left(P_{2}^{\prime }-P_{2}\right)>0.$$

As discussed earlier, with one gap shrinking and the other expanding, only Case (c)–or the majorization relation—holds ($P_{3}>P_{3}^{\prime },\;\;P_{1}<P_{1}^{\prime }$). PWC then necessitates a definite relation between $P_{2}$ and $P_{2}^{\prime }$ (which is otherwise not guaranteed by the majorization condition). Thus, G-2 implies $P_{2}>P_{2}^{\prime }$, while G-3 implies $P_{2}<P_{2}^{\prime }$. Thus, along with the majorization condition, a definite inequality between the middle level probabilities determines the PWC when the total gap is held fixed during the cycle.

### 3.3. Constraints on the Reservoir Temperatures

In the following, we show that further insight can be gained if the situation is analyzed in terms of the conditions on the reservoir temperatures. Suppose the total gap *expands*, i.e., $\omega_{1}^{\prime }+\omega_{2}^{\prime }>\omega_{1}+\omega_{2}$, or $\omega^{\prime }>\omega$. From the above analysis, we note that one gap must expand while the other shrinks. In other words, G-2 and G-3 are applicable, and accordingly, we can say that majorization becomes a *necessary* condition for the engine (Case (c)). Thus, Equation (10) is applicable, and can be rewritten as:

(14) $$T_{1}>T_{2}\left(\frac{\omega }{\omega^{\prime }}\right).$$

However, as we have assumed $T_{1}>T_{2}$, Equation (14) provides a weaker condition if $\omega^{\prime }>\omega$. Note that Case (c) does not pre-fix a relation between $P_{2}$ and $P_{2}^{\prime }$. However, for the total gap expanding, one of the following two scenarios applies. For G-2, $P_{2}>P_{2}^{\prime }$ is a necessary criterion for PWC, which can be seen by writing Equation (7) in the following form:

(15) $$W=\left(\omega -\omega^{\prime }\right)\left(P_{3}-P_{3}^{\prime }\right)+\left(\omega_{1}-\omega_{1}^{\prime }\right)\left(P_{2}-P_{2}^{\prime }\right).$$

Now, G-2 implies $\omega_{1}>\omega_{1}^{\prime }$. Therefore, with $P_{3}>P_{3}^{\prime }$ (due to majorization) and $\omega <\omega^{\prime }$ (total gap expanding), we must have $P_{2}>P_{2}^{\prime }$ to ensure PWC. Then, along with the other inequality for majorization $P_{1}<P_{1}^{\prime }$, we obtain $T_{1}>T_{2}\left(\omega_{1}/\omega_{1}^{\prime }\right)$ as a stronger condition on the reservoir temperatures. Similarly, it follows from Equation (15) that for G-3 (implying $\omega_{1}<\omega_{1}^{\prime }$), $P_{2}<P_{2}^{\prime }$ is a necessary criterion for PWC. Along with the inequality $P_{3}>P_{3}^{\prime }$, this yields a stronger condition on the temperatures as $T_{1}>T_{2}\left(\omega_{2}/\omega_{2}^{\prime }\right)$.

Alternatively, the total gap may *shrink*, i.e., $\omega^{\prime }<\omega$, which certainly holds when both gaps shrink (G-1), and is also valid for Cases (a), (b), or (c). The corresponding conditions on reservoir temperatures can be derived from the previous analysis, and have been summarized in Table 1.

Furthermore, the total gap may shrink with G-2 and G-3 as well. Then, only Case (c) is valid, i.e., Equation (14) holds. Here, each of G-2 and G-3 allows both possible relations between $P_{2}$ and $P_{2}^{\prime }$, as depicted in Table 2.

### 3.4. Examples

**Figure 3:**
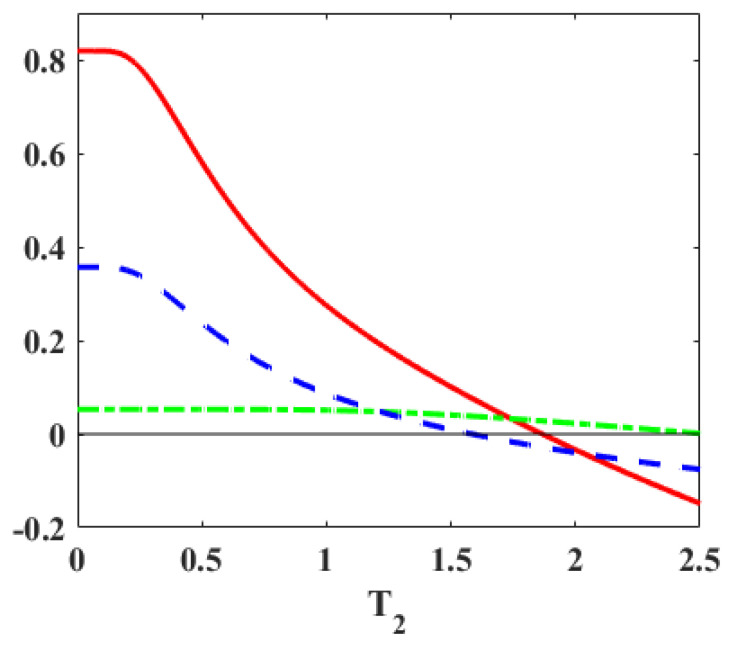
The solid curve (red online) represents the work per cycle vs. the temperature $T_{2}$ of the cold reservoir. The parameters are set at $T_{1}=4$, J=2, $B_{1}=5$, and $B_{2}=3$. With $B_{1}>B_{2}>0$ and *J* fixed, both gaps are shrinking. The dashed (blue) and dot-dashed (green) curves denote $\left(P_{1}^{\prime }-P_{1}\right)$ and $\left(P_{3}-P_{3}^{\prime }\right)$, respectively. The majorization relation ($P≺P^{\prime }$) holds if both dashed and dot-dashed curves are positive. It is seen that majorization is a sufficient, but not a necessary, condition for the work to be positive.

To illustrate our analytic observations, we employ a toy Hamiltonian with the energy spectrum at the hot and cold thermalization stages as ($\varepsilon_{3}=B_{1},\varepsilon_{2}=-J,\varepsilon_{1}=-B_{1})$, and $\left(\varepsilon_{3}^{\prime }=B_{2},\varepsilon_{2}^{\prime }=-J,\varepsilon_{1}^{\prime }=-B_{2}\right)$, respectively. With $B_{1}>B_{2}>0$ and a fixed *J* value, it allows us to shrink both the gaps in the first adiabatic stage. In Figure 3, we observe that work may be positive whether majorization holds or not. As the blue curve crosses the zero line ($P_{1}^{\prime }-P_{1}<0$), the majorization condition is violated, but the work is still positive. Thus, majorization is not a necessary condition for PWC when both gaps are shrinking. However, if the majorization holds ($P≺P^{\prime }$), then work is positive, thus making the majorization relation a sufficient condition for the Otto engine.

Further, with parameters of Figure 3 at $T_{1}=4,J=2,B_{1}=5$ and $B_{2}=3$, we have $\omega_{1}=3,\omega_{2}=7,\omega =10$ and $\omega_{1}^{\prime }=1,\omega_{2}^{\prime }=5,\omega^{\prime }=6$, where energies are measured in units of temperature. Thus, both gaps are shrinking here (G-1). Case (c) is a sufficient condition for PWC, and from Table 1, we have the condition $T_{2}<T_{1}\left(\omega^{\prime }/\omega \right)=2.4$. When the blue line crosses the zero mark, a transition happens to Case (a) which is explained in more detail in Section 4.

**Figure 4:**
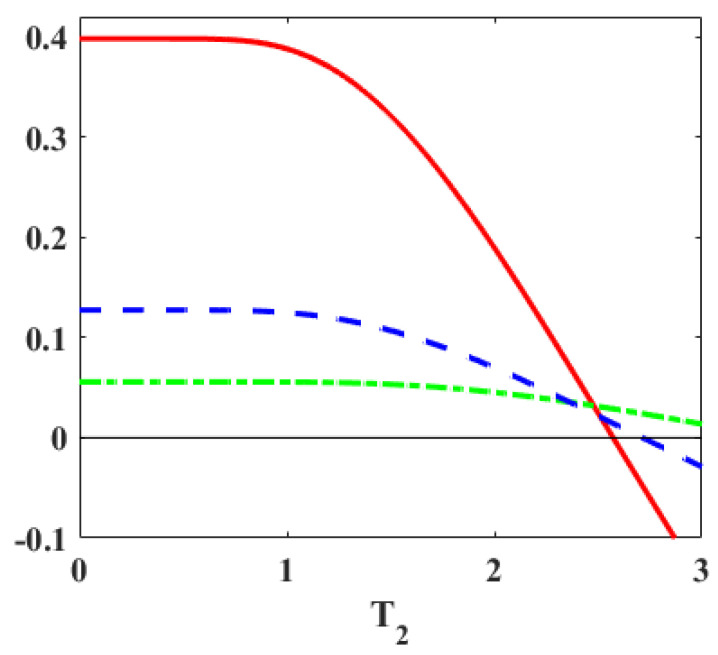
The red line denotes the work outout, while the dashed (blue) and dot-dashed (green) curves denote $\left(P_{1}^{\prime }-P_{1}\right)$ and $\left(P_{3}-P_{3}^{\prime }\right)$ respectively, for the energy spectrum as [($\varepsilon_{3}=J,\varepsilon_{2}=B_{1},$$\varepsilon_{1}=-B_{1}),\;(\varepsilon_{3}^{\prime }=J,\varepsilon_{2}^{\prime }=B_{2},\varepsilon_{1}^{\prime }=-B_{2}$)]. The parameters are set at $T_{1}=4,J=6,B_{1}=5,B_{2}=3$. With $B_{1}>B_{2}>0$ and *J* fixed, the lower gap shrinks while the upper one expands. The work is positive only if the majorization condition ($P≺P^{\prime }$) is satisfied. If majorization does not hold, then work is negative.

In Figure 4, the energy spectrum is chosen as ($\varepsilon_{3}=J,\varepsilon_{2}=B_{1},\varepsilon_{1}=-B_{1}$) and ($\varepsilon_{3}^{\prime }=J,\varepsilon_{2}^{\prime }=B_{2},\varepsilon_{1}^{\prime }=-B_{2}$). With $B_{1}>B_{2}>0$ and fixed *J*, the lower gap shrinks while the upper one expands. As we have demonstrated analytically, majorization is a necessary, but not a sufficient, condition for positive work extraction in this scenario. This is illustrated in Figure 4 by the fact that the green and blue curves for majorization may stay positive even though the work output becomes negative. Figure 4 corresponds to the G-2 scenario, where majorization is a necessary condition for work extraction. Also, when the blue line is above the green line, we have $P_{1}^{\prime }-P_{1}>P_{3}-P_{3}^{\prime }$, implying $P_{2}>P_{2}^{\prime }$. Thus, from Table 2, the constraint on temperature is $T_{2}<T_{1}\left(\omega_{1}^{\prime }/\omega_{1}\right)=2.4$. So, a stronger bound for $T_{2}$ is obtained for work extraction from Table 2 since the inequality $T_{2}<T_{1}\left(\omega^{\prime }/\omega \right)$ following from the majorization condition only yields $T_{2}<3.2$.

**Figure 5:**
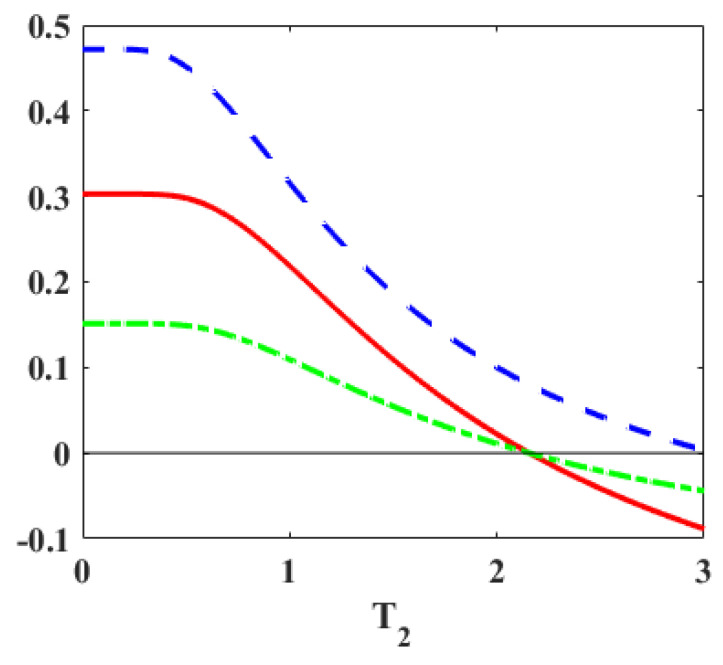
The red line denotes the work outout, while the dashed (blue) and dot-dashed (green) curves denote $\left(P_{1}^{\prime }-P_{1}\right)$ and $\left(P_{3}-P_{3}^{\prime }\right)$ respectively, where the energy spectrum chosen is ($\varepsilon_{3}=J_{1},\varepsilon_{2}=B,\varepsilon_{1}=-B$) and ($\varepsilon_{3}^{\prime }=J_{2},\varepsilon_{2}^{\prime }=B,\varepsilon_{1}^{\prime }=-B$). With constant B>0 and $J_{1}>J_{2}>0$, the lower energy gap is kept fixed while the upper gap shrinks. The parameters used are $T_{1}=4,B=1,J_{1}=4,J_{2}=2$. The machine works as an engine so long as the majorization condition is obeyed. As the green line crosses the zero mark, the work becomes negative.

**Figure 6:**
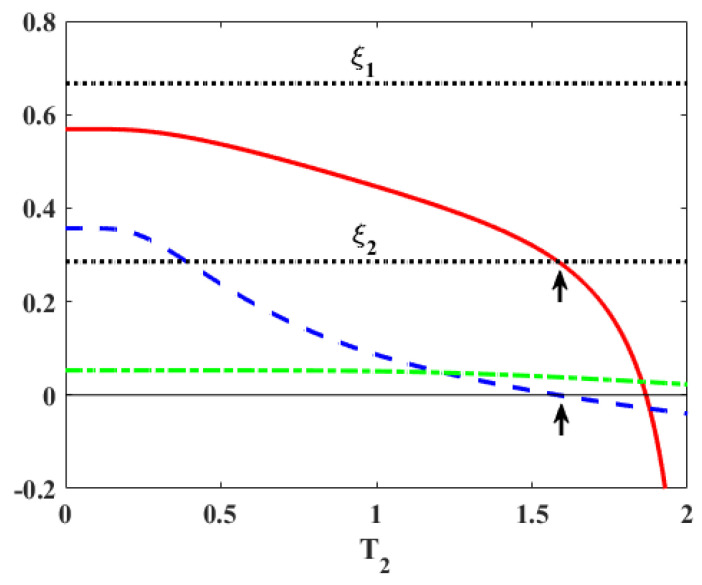
The solid curve (red online) represents the efficiency of the Otto cycle while the dotted horizontal lines corresponds to $\xi_{i}\equiv \left(\omega_{i}-\omega_{i}^{\prime }\right)/\omega_{i}$ for i=1,2. The energy spectra and the parameters are chosen as for Figure 3, so that both gaps are shrinking and $\omega_{2}^{\prime }/\omega_{2}>\omega_{1}^{\prime }/\omega_{1}$. As explained in the Section 4, Case (c) applies between the bounds given by dotted lines (Equation (21)) while below the lower bound $\xi_{2}$, Case (a) is applicable (Equation (19)). The transition (upper arrow) from Case (c) to Case (a) happens when $P_{1}^{\prime }-P_{1}$ (dotted, blue) changes sign from positive to negative (lower arrow). $P_{3}-P_{3}^{\prime }$ (dot-dashed, green) remains positive.

Similarly, with one gap fixed, as majorization provides a necessary and sufficient condition for the engine, if the majorization is violated, the work becomes negative, as shown in Figure 5. Finally, in Figure 3, for extremely low cold bath temperatures, the work (as well as the efficiency in Figure 6) exhibits a plateau versus the temperature $T_{2}$. The reason for this behavior is that $P_{1}^{\prime }\approx 1$ and $P_{3}^{\prime }\approx 0$ in this range of temperatures and increasing $T_{2}$ does not lead to enough excitations to change these probabilities. As a result, heat and work (Equations (5) and (7)) remain constant, and thereby the efficiency also shows a plateau as in Figure 6.

To summarize our results so far, if both energy gaps in a 3LS shrink during the first adiabatic process, then any one of Cases (a), (b), and (c) may apply. If one of the gaps shrinks while the other expands, then the majorization relation ($P≺P^{\prime }$) *must* hold to allow the machine to perform as an engine. Note that when both energy gaps are expanding, the same majorization relation holds, but PWC does not. Therefore, G-4 is forbidden for an Otto engine.

## 4. Otto Efficiency

A two-level Otto engine has but one energy gap, which is modulated during the cycle ($\omega_{0}↔\omega_{0}^{\prime }$). The efficiency of this cycle is $\left(1-\omega_{0}^{\prime }/\omega_{0}\right)$, where $\omega_{0}^{\prime }<\omega_{0}$ [73]. On the other hand, a 3LS has two gaps and thus offers greater flexibility. After studying the PWC, we look at the efficiency of the cycle, $\eta =W/Q_{1}$, which is given by

(16) $$\begin{array}{cc} \eta & =\frac{\left(P_{3}-P_{3}^{\prime }\right)\left(\omega_{2}-\omega_{2}^{\prime }\right)+\left(P_{1}^{\prime }-P_{1}\right)\left(\omega_{1}-\omega_{1}^{\prime }\right)}{\left(P_{3}-P_{3}^{\prime }\right)\omega_{2}+\left(P_{1}^{\prime }-P_{1}\right)\omega_{1}}. \end{array}$$

Let us consider Case (a) for which both gaps are shrinking and denote $\xi_{i}\equiv \left(\omega_{i}-\omega_{i}^{\prime }\right)/\omega_{i}>0$, where i=1,2. For this case, we express the efficiency in the following alternate forms:

(17) $$\begin{array}{cc} \eta_{\left(a\right)} & =\xi_{1}\left[\frac{\frac{\omega_{2}-\omega_{2}^{\prime }}{\omega_{1}-\omega_{1}^{\prime }}-\frac{P_{1}-P_{1}^{\prime }}{P_{3}-P_{3}^{\prime }}}{\frac{\omega_{2}}{\omega_{1}}-\frac{P_{1}-P_{1}^{\prime }}{P_{3}-P_{3}^{\prime }}}\right], \end{array}$$

(18) $$\begin{array}{cc} \; & =\xi_{2}\left[\frac{\frac{P_{3}-P_{3}^{\prime }}{P_{1}-P_{1}^{\prime }}-\frac{\omega_{1}-\omega_{1}^{\prime }}{\omega_{2}-\omega_{2}^{\prime }}}{\frac{P_{3}-P_{3}^{\prime }}{P_{1}-P_{1}^{\prime }}-\frac{\omega_{1}}{\omega_{2}}}\right]. \end{array}$$

Due to Equation (8), we have $\xi_{2}<\xi_{1}$. Further, the factors enclosed in the square parentheses above are less than unity. Thus, we infer that for Case (a), the efficiency is bounded as:

(19) $$\eta_{\left(a\right)}<\xi_{2}<\xi_{1}.$$

Along similar lines, we can show that Case (b) and G-(1) imply:

(20) $$\eta_{\left(b\right)}<\xi_{1}<\xi_{2}.$$

Note that for Case (b), $\xi_{1}<\xi_{2}$ holds (see Equation (9)).

Next, we consider Case (c), where the majorization relation holds ($P≺P^{\prime }$). To make a comparison with other cases, let us assume that Case (a) applies, so that $\omega_{2}/\omega_{1}<\omega_{2}^{\prime }/\omega_{1}^{\prime }$. Then, we can show

(21) $$\xi_{2}<\eta_{\left(c\right)}<\xi_{1}.$$

Compared with Equation (19), we conclude that majorization or Case (c) provides a higher efficiency than Case (a). As shown in Figure 6, within the bounds, Case (c) applies. When the efficiency breaches the lower bound, Case (a) is applicable.

Similarly, with both gaps shrinking and Case (b) applicable, we have $\omega_{2}/\omega_{1}>\omega_{2}^{\prime }/\omega_{1}^{\prime }$. Then, we can derive $\xi_{2}>\eta_{\left(c\right)}>\xi_{1}$. Thus, again, Case (c) yields a higher efficiency than Case (b). Therefore, we may conclude that efficiency enhancement can be achieved with the majorization relation when both gaps are shrinking. On the other hand, with one of the gaps shrinking, only the majorization relation holds. If the lower gap shrinks, we obtain $\eta_{\left(c\right)}<\xi_{1}$, while if the upper gap shrinks, we have $\eta_{\left(c\right)}<\xi_{2}$. In the special case where one of the energy gaps is fixed, the efficiency is bounded from above by $\xi_{i}$, denoting the variable gap.

## 5. Swap Engine with Two Three-Level Systems

The quantum Otto cycle can be easily implemented when the 3LS is a spin-1 particle or a qutrit where the two energy gaps are equal, and they can be shrunk or expanded by controlling the external magnetic field. However, when the gaps are unequal or have to be modulated in an opposing manner, it may require a more elaborate control of external fields [129]. Under such circumstances, an alternate model of work extraction may be more practical, which yields the same expressions for heat and work as the Otto cycle treated above. Following Ref. [8], we consider a two-stroke heat engine with two 3LSs constituting the working medium and the Hamiltonian given by: $H=H_{1}⊗I+I⊗H_{2}$, where The respective Hamiltonian of each 3LS is given by:

(22) $$H_{1}=\sum_{j=1}^{3}\varepsilon_{j}|\psi_{j}\rangle \langle \psi_{j}|,\;H_{2}=\sum_{j=1}^{3}\varepsilon_{j}^{\prime }|\phi_{j}\rangle \langle \phi_{j}|,$$

with the energy eigenvalues ordered as: $\varepsilon_{1}<\varepsilon_{2}<\varepsilon_{3}$ ($\varepsilon_{1}^{\prime }<\varepsilon_{2}^{\prime }<\varepsilon_{3}^{\prime }$). Note that $\varepsilon_{j}$ ($\varepsilon_{j}^{\prime }$) correspond to energy levels at the beginning (end) of the first adiabatic process of the Otto cycle described in Section 3. Thereby, the corresponding gaps $\omega_{j}$ and $\omega_{j}^{\prime }$ are also similar to those in the Otto cycle.

Now, the initial state of each 3LS is a thermal state in equilibrium with a hot and a cold reservoir, respectively: $\rho_{1}=\exp\left(-H_{1}/T_{1}\right)/Z_{1}$ and $\rho_{2}=\exp\left(-H_{2}/T_{2}\right)/Z_{2}$, where $T_{1}>T_{2}$. The partition function $Z_{i}=\mathrm{Tr}\left[e^{-H_{i}/T_{i}}\right]$, for i=1,2. So, with $\rho_{1}⊗\rho_{2}$ as the initial state of the working medium, the two systems are allowed to interact via a time-dependent interaction $V\left(t\right)=\kappa \left(e^{-iHt/\hbar }\;S\;e^{iHt/\hbar }\right)$ for a finite time τ [8], which is switched ON and then turned OFF after time τ. Here, parameter κ represents the strength of interaction and *S* denotes the swap operation, acting on the bipartite state as $S(|\psi_{j}\rangle ⊗|\phi_{k}\rangle )=|\phi_{k}\rangle ⊗|\psi_{j}\rangle$. The total Hamiltonian H+V(t) undergoes a cyclic evolution in this period. The final state of the working medium is given by: $\rho_{1}^{\prime }⊗\rho_{2}^{\prime }=\rho_{2}⊗\rho_{1}$. The swap operation leads to a lower total mean energy, the difference being extracted as work in this step, given by

(23) $$W=\mathrm{Tr}\left[\left(\rho_{1}⊗\rho_{2}\right)H\right]-\mathrm{Tr}\left[\left(\rho_{2}⊗\rho_{1}\right)H\right]=\sum_{k}\left(\varepsilon_{k}-\varepsilon_{k}^{\prime }\right)\left(P_{k}-P_{k}^{\prime }\right).$$

The interaction strength is assumed to be sufficiently strong so as to neglect the influence of the heat reservoirs, and the first stroke is regarded as thermally isolated. This strength is fundamentally limited by the relaxation time of the quantum system, which varies across different experimental platforms. For high-fidelity swap operations, the interaction time must remain well below the relaxation times.

In the second step, the systems return to their initial states upon thermalization with their respective reservoirs, thus completing a two-step cycle. The heat exchange during the second step with each reservoir is given by:

(24) $$\begin{array}{cc} Q_{1} & =\mathrm{Tr}\left[\left(\rho_{1}-\rho_{1}^{\prime }\right)H_{1}\right]=\sum_{k}\varepsilon_{k}\left(P_{k}-P_{k}^{\prime }\right), \end{array}$$

(25) $$\begin{array}{cc} Q_{2} & =\mathrm{Tr}\left[\left(\rho_{2}-\rho_{2}^{\prime }\right)H_{2}\right]=\sum_{k}\varepsilon_{k}^{\prime }\left(P_{k}^{\prime }-P_{k}\right), \end{array}$$

where $P_{k}\left(P_{k}^{\prime }\right)$ denote the canonical probabilities of the 3LS in equilibrium with the hot (cold) reservoir. From energy conservation, the net work extracted per cycle is given by $W=Q_{1}+Q_{2}$. It is clear that these expressions for heat and work match with Equations (1) and (2) for the Otto cycle with one 3LS. Therefore, the analysis undertaken in this paper, with regard to PWC and so on, becomes directly applicable to the above swap engine. However, it may be added that even if the swap engine based on two qubits has been well studied [130], a qudits-based swap engine has been recently investigated [131]. Note that a swap engine, in general, does not lead to a passive state or the work extracted being less than the ergotropy of the initial state. In contrast, for a two-qubit system, the swap operation leads to the extraction of ergotropy [8]. Finally, to the best of the authors’ knowledge, the design of appropriate gate operations for a quantum information processor to implement the swap operation on a pair of generic three-level systems is an open problem.

## 6. Conclusions

We have analyzed the mutually compatible changes in the energy gaps and the thermal distributions in a multilevel-based quantum Otto cycle. The thermodynamic quantities specifying engine operation, such as work performed and heat exchange, for an *n*-level quantum Otto engine are derived within the framework of majorization theory. Our analysis shows that when the energy gaps during the first adiabatic stroke are non-increasing, majorization serves as a sufficient criterion for positive work extraction. However, in the special case where all the energy gaps change by the same ratio, majorization provides both necessary and sufficient criteria. Additionally, majorization determines the correct direction of heat exchange required for engine operation. A detailed analysis of the three-level system reveals that, first and foremost, it is essential for at least one energy gap to shrink in the first adiabatic stage of the given cycle to achieve the operation as an Otto engine. When both gaps expand, net work cannot be extracted. Second, when both gaps shrink, the majorization condition $\left(P≺P^{\prime }\right)$ serves as a sufficient criterion to obtain an engine. Interestingly, majorization becomes a necessary condition when one gap shrinks while the other expands. We have studied the effect of changes in the energy gaps and how they determine the constraints on the reservoir temperatures for given gap values. It is also observed that for both gaps shrinking, Otto efficiency can be enhanced for Case (c) compared to Cases (a) and (b). In the special case where one of the two gaps stays fixed during the cycle, the validity of the majorization relation is the only possible scenario in favor of an engine, making majorization a necessary and sufficient condition.

The present work improves upon the analysis of ref. [127], which was restricted to the high temperature limit and mainly focused on the Gtion of both gaps shrinking. A classical heat engine requires the two heat reservoirs to be at different temperatures ($T_{1}\neq T_{2}$), while an Otto engine based on a two-level system imposes a stronger condition: $T_{1}>T_{2}(\omega /\omega^{\prime }$). Reference [127] discussed that, for a 3LS with both gaps shrinking, the corresponding condition can be looser than in a two-level system. Our analysis has comprehensively determined the constraints on the reservoir temperatures for the 3LS across all possible changes in the energy gaps. Interestingly, we notice that when one of the gaps is shrinking and the other is expanding, while majorization is a necessary condition for PWC, a stronger constraint on the reservoir temperatures may be obtained based on the inequality between $P_{2}$ and $P_{2}^{\prime }$. Thus, our results provide a benchmark for characterizing the performance of a 3-level Otto engine, which can be useful for making comparisons with more sophisticated Otto engine models, such as those including the effects of finite time, incomplete thermalization, coherence and so on. Also, the analysis can be applied to a swap engine based on two 3LSs, since the quasi-static quantum Otto engine can be mapped to the swap engine. It will be interesting to perform this analysis in other generic settings, for example, when the usual equilibrium reservoirs are replaced by squeezed or engineered reservoirs, and to determine the corresponding operating conditions. This will help establish the scope and limitations of the concept of majorization in these settings.

## Figures and Tables

**Table 1:** Conditions on the reservoir temperatures when both gaps shrink (G-1).

	G-1
Case (a)	$T_{2}\left(\omega_{2}/\omega_{2}^{\prime }\right)<T_{1}<T_{2}\left(\omega_{1}/\omega_{1}^{\prime }\right)$
Case (b)	$T_{2}\left(\omega_{1}/\omega_{1}^{\prime }\right)<T_{1}<T_{2}\left(\omega_{2}/\omega_{2}^{\prime }\right)$
Case (c)	$T_{1}>T_{2}\left(\omega /\omega^{\prime }\right)$

**Table 2:** Conditions on the reservoir temperatures if the total gap shrinks while one of the gaps expands. The majorization relation (Case (c)) only provides a necessary criterion for PWC.

Case (c)	G-2	G-3
$P_{2}<P_{2}^{\prime }$	$T_{1}>T_{2}\left(\omega /\omega^{\prime }\right)$	$T_{1}>T_{2}\left(\omega_{2}/\omega_{2}^{\prime }\right)$
$P_{2}>P_{2}^{\prime }$	$T_{1}>T_{2}\left(\omega_{1}/\omega_{1}^{\prime }\right)$	$T_{1}>T_{2}\left(\omega /\omega^{\prime }\right)$

## Data Availability

All data used for analysis has been provided in the Figures.

## Appendix A

### Appendix A.1. n-Level System

We derive Equations (3) and (4) from the main text. Expanding Equation (1) and applying normalization conditions $P_{1}=1-\sum_{k=2}^{n}P_{k}$ and $P_{1}^{\prime }=1-\sum_{k=2}^{n}P_{k}^{\prime }$, we derive

(A1) $$\begin{array}{cc} Q_{1} & =\varepsilon_{1}\left[\left(P_{n}^{\prime }+P_{n-1}^{\prime }\ldots +P_{2}^{\prime }\right)-\left(P_{n}+P_{n-1}+\ldots +P_{2}\right)\right]+\varepsilon_{2}\left(P_{2}-P_{2}^{\prime }\right)\ldots +\varepsilon_{n}\left(P_{n}-P_{n}^{\prime }\right)\\ \; & =\left(\varepsilon_{n}-\varepsilon_{1}\right)\left(P_{n}-P_{n}^{\prime }\right)+\left(\varepsilon_{n-1}-\varepsilon_{1}\right)\left(P_{n-1}-P_{n-1}^{\prime }\right)+\ldots +\left(\varepsilon_{2}-\varepsilon_{1}\right)\left(P_{2}-P_{2}^{\prime }\right)\\ \; & =\left(\omega_{1}+\omega_{2}+\ldots +\omega_{n-1}\right)\left(P_{n}-P_{n}^{\prime }\right)+\left(\omega_{1}+\omega_{2}+\ldots +\omega_{n-2}\right)\left(P_{n-1}-P_{n-1}^{\prime }\right)+\ldots \\ \; & +\omega_{1}\left(P_{2}-P_{2}^{\prime }\right)\\ \; & =\omega_{1}\left[\left(P_{n}+P_{n-1}+\ldots +P_{2}\right)-\left(P_{n}^{\prime }+P_{n-1}^{\prime }+\ldots +P_{2}^{\prime }\right)\right]\\ & +\omega_{2}\left[\left(P_{n}+P_{n-1}+\ldots +P_{3}\right)-\left(P_{n}^{\prime }+P_{n-1}^{\prime }+\ldots +P_{3}\right)\right]+\ldots +\omega_{n-1}\left(P_{n}-P_{n}^{\prime }\right)\\ \; & =\sum_{j=1}^{n-1}\omega_{j}M_{j}, \end{array}$$

where $M_{j}=\sum_{i=j+1}^{n}\left(P_{i}-P_{i}^{\prime }\right),\;\;\;j=1,2,\ldots ,\left(n-1\right).$

Similarly, we obtain

(A2) $$Q_{2}=\sum_{j=1}^{n-1}-\omega_{j}^{\prime }M_{j}.$$

The expression for net work for an *n*-level system can be obtained from $W=Q_{1}+Q_{2}$ as follows:

(A3) $$\begin{array}{cc} W & =\sum_{j=1}^{n-1}\left(\omega_{j}-\omega_{j}^{\prime }\right)M_{j}. \end{array}$$

The motivation for writing Equations (A1)–(A3) in terms of $M_{j}$ originates from the theory of majorization. For two probability distributions arranged in decreasing order, the majorization relation satisfies the following conditions:

(A4) $$\begin{array}{cc} P≺P^{\prime }⟹ & \sum_{i=j+1}^{n}\left(P_{i}-P_{i}^{\prime }\right)\geq 0\equiv M_{j}\geq 0,\;\forall \;j=1,2,\ldots \left(n-1\right), \end{array}$$

along with $\sum_{i=1}^{n}P_{i}=\sum_{i=1}^{n}P_{i}^{\prime }=1$. Now, solving the majorization inequalities,

$$\begin{array}{cc} M_{j} & =\sum_{i=j+1}^{n}P_{i}-P_{i}^{\prime },\;\forall \;j=1,2,...\left(n-1\right)\\ \; & =\left(P_{j+1}+P_{j+2}+\ldots +P_{n}\right)-\left(P_{j+1}^{\prime }+P_{J+2}^{\prime }+\ldots +P_{n}^{\prime }\right), \end{array}$$

solving the first bracket by substituting the explicit form of the probability distribution,

(A5) $$\begin{array}{cc} \; & \;\frac{e^{-\varepsilon_{j+1}/T_{1}}+e^{-\varepsilon_{j+2}/T_{1}}+\ldots +e^{-\varepsilon_{n}/T_{1}}}{e^{-\varepsilon_{1}/T_{1}}+e^{-\varepsilon_{2}/T_{1}}+\ldots +e^{-\varepsilon_{n}/T_{1}}},\\ \; & \frac{1+e^{-\left(\varepsilon_{j+2}-\varepsilon_{j+1}\right)/T_{1}}+\ldots +e^{-\left(\varepsilon_{n}-\varepsilon_{j+1}\right)/T_{1}}}{e^{\left(\varepsilon_{j+1}-\varepsilon_{1}\right)/T_{1}}+e^{\left(\varepsilon_{j+1}-\varepsilon_{2}\right)/T_{1}}+\ldots +1+e^{-\left(\varepsilon_{j+2}-\varepsilon_{j+1}\right)/T_{1}}+\ldots +e^{-\left(\varepsilon_{n}-\varepsilon_{j+1}\right)/T_{1}}},\\ \; & \frac{1+e^{-\omega_{j+1}/T_{1}}+\ldots +e^{-\left(\sum_{k=j+1}^{n-1}\omega_{k}\right)/T_{1}}}{e^{\left(\sum_{k=1}^{j}\omega_{k}\right)/T_{1}}+e^{\left(\sum_{k=2}^{j}\omega_{k}\right)/T_{1}}+\ldots +e^{\omega_{j}/T_{1}}+1+e^{-\omega_{j+1}/T_{1}}+\ldots +e^{-\left(\sum_{k=j+1}^{n-1}\omega_{k}\right)/T_{1}}},\\ \; & \;\frac{1}{\left(\frac{e^{\left(\sum_{k=1}^{j}\omega_{k}\right)/T_{1}}+e^{\left(\sum_{k=2}^{j}\omega_{k}\right)/T_{1}}+\ldots +e^{\omega_{j}/T_{1}}}{1+e^{-\omega_{j+1}/T_{1}}+e^{-\omega_{j+2}/T_{1}}+\ldots +e^{-\left(\sum_{k=j+1}^{n-1}\omega_{k}\right)/T_{1}}}\right)+1}. \end{array}$$

Similarly, for the second bracket, we obtain

(A6) $$\frac{1}{\left(\frac{e^{\left(\sum_{k=1}^{j}\omega_{k}^{\prime }\right)/T_{2}}+e^{\left(\sum_{k=2}^{j}\omega_{k}^{\prime }\right)/T_{2}}+\ldots +e^{\omega_{j}^{\prime }/T_{2}}}{1+e^{-\omega_{j+1}^{\prime }/T_{2}}+e^{-\omega_{j+2}^{\prime }/T_{2}}+\ldots +e^{-\left(\sum_{k=j+1}^{n-1}\omega_{k}^{\prime }\right)/T_{2}}}\right)+1}.$$

From Equations (A5) and (A6), we observe that the condition $\frac{\omega_{j}}{T_{1}}<\frac{\omega_{j}^{\prime }}{T_{2}}$ for all j=1,2,…,(n−1) leads to the inequality $\frac{\sum_{k=1}^{m}\omega_{k}}{T_{1}}<\frac{\sum_{k=1}^{m}\omega_{k}^{\prime }}{T_{2}}$ for all m=1,2,…,(n−1). This ensures that all majorization relations are satisfied. Consequently, a sufficient condition for positive work extraction (Equation (A3)) is given by $\omega_{j}^{\prime }\leq \omega_{j}\leq \frac{T_{1}}{T_{2}}\omega_{j}^{\prime }$ for all j=1,2,…,(n−1) in the *n*-level system.

For the special case where all the gaps are changed by the same ratio, i.e., $\frac{\omega_{1}}{\omega_{1}^{\prime }}=\frac{\omega_{2}}{\omega_{2}^{\prime }}=\ldots =\frac{\omega_{n-1}}{\omega_{n-1}^{\prime }}$, the expression of work is simplified to

(A7) $$\begin{array}{cc} W & =\left(1-\frac{\omega_{k}^{\prime }}{\omega_{k}}\right)\sum_{j=1}^{n-1}\omega_{j}M_{j},\\ \; & =\sum_{j=1}^{n-1}\eta \omega_{j}M_{j}, \end{array}$$

where $\eta =W/Q_{1}=1-\frac{\omega_{k}^{\prime }}{\omega_{k}}$ for all k=1,2,…,(n−1). The majorization relation ensures both positive work and heat exchange. The efficiency, given by η, is bounded by the Carnot limit according to the second law $1-\frac{\omega_{k}^{\prime }}{\omega_{k}}<1-\frac{T_{2}}{T_{1}}$, which implies $\frac{\omega_{k}}{T_{1}}<\frac{\omega_{k}^{\prime }}{T_{2}}$ for all k=1,2,…,(n−1). This guarantees that the majorization relations, as expressed in Equations (A5) and (A6), hold. Therefore, when all the energy gaps change by the same ratio, the majorization relation becomes both a necessary and sufficient criterion for engine operation.

### Appendix A.2. Case (a)

Case (a) specifies $P_{1}>P_{1}^{\prime }$, $P_{3}>P_{3}^{\prime }$ and $P_{2}<P_{2}^{\prime }$. By using the explicit forms of the probability distributions given in Section 3, we obtain

(A8) $$\frac{P_{1}}{P_{2}}>\frac{P_{1}^{\prime }}{P_{2}^{\prime }}\Rightarrow \frac{\omega_{1}}{T_{1}}>\frac{\omega_{1}^{\prime }}{T_{2}}.$$

Since $T_{1}>T_{2}>0$, we deduce that $\omega_{1}>\omega_{1}^{\prime }$. This implies that the lower gap must shrink in the first adiabatic step. The engine operating conditions, as applied to Equations (5)–(7), imply the following inequalities:

(A9) $$\begin{array}{c} Q_{1}>0\Rightarrow \frac{\omega_{2}}{\omega_{1}}>\frac{P_{1}-P_{1}^{\prime }}{P_{3}-P_{3}^{\prime }}, \end{array}$$

(A10) $$\begin{array}{c} Q_{2}<0\Rightarrow \frac{\omega_{2}^{\prime }}{\omega_{1}^{\prime }}>\frac{P_{1}-P_{1}^{\prime }}{P_{3}-P_{3}^{\prime }}, \end{array}$$

(A11) $$\begin{array}{c} W>0\Rightarrow \frac{\omega_{2}-\omega_{2}^{\prime }}{\omega_{1}-\omega_{1}^{\prime }}>\frac{P_{1}-P_{1}^{\prime }}{P_{3}-P_{3}^{\prime }}. \end{array}$$

Given that the right-hand side of Equation (A11) is a positive quantity, we have

(A12) $$\begin{array}{cc} \frac{\omega_{2}-\omega_{2}^{\prime }}{\omega_{1}-\omega_{1}^{\prime }} & >0, \end{array}$$

implying that $\omega_{2}>\omega_{2}^{\prime }$, as well. Thus, Case (a) requires that both gaps must shrink in the first adiabatic step. Additionally, we have

(A13) $$\frac{P_{3}}{P_{2}}>\frac{P_{3}^{\prime }}{P_{2}^{\prime }}\Rightarrow \frac{\omega_{2}}{T_{1}}<\frac{\omega_{2}^{\prime }}{T_{2}}.$$

Thus, Case (a) also implies that the the upper gap is constrained as follows:

(A14) $$\begin{array}{cc} \frac{T_{1}}{T_{2}}\omega_{2}^{\prime } & >\omega_{2}>\omega_{2}^{\prime }. \end{array}$$

From Equations (A8) and (A13), we deduce that

(A15) $$\frac{\omega_{2}^{\prime }}{\omega_{1}^{\prime }}>\frac{\omega_{2}}{\omega_{1}}.$$

Using (A11), (A15) and the mediant inequality [132], we can write

(A16) $$\frac{\omega_{2}^{\prime }}{\omega_{1}^{\prime }}>\frac{\omega_{2}}{\omega_{1}}>\frac{\omega_{2}-\omega_{2}^{\prime }}{\omega_{1}-\omega_{1}^{\prime }}>\frac{P_{1}-P_{1}^{\prime }}{P_{3}-P_{3}^{\prime }}.$$

### Appendix A.3. Case (b)

Case (b) specifies $P_{1}<P_{1}^{\prime }$, $P_{3}<P_{3}^{\prime }$, and $P_{2}>P_{2}^{\prime }$. From the explicit form of the probability distributions, we obtain

(A17) $$\frac{P_{3}}{P_{2}}<\frac{P_{3}^{\prime }}{P_{2}^{\prime }}\Rightarrow \frac{\omega_{2}}{T_{1}}>\frac{\omega_{2}^{\prime }}{T_{2}}.$$

Since $T_{1}>T_{2}>0$, Equation (A17) holds if $\omega_{2}>\omega_{2}^{\prime }$. Thus, the upper gap should shrink in the first adiabatic step for Case (b).

From Equations (5)–(7), we can write

(A18) $$\begin{array}{cc} Q_{1}>0 & \Rightarrow \frac{\omega_{1}}{\omega_{2}}>\frac{P_{3}^{\prime }-P_{3}}{P_{1}^{\prime }-P_{1}}, \end{array}$$

(A19) $$\begin{array}{cc} Q_{2}<0 & \Rightarrow \frac{\omega_{1}^{\prime }}{\omega_{2}^{\prime }}>\frac{P_{3}^{\prime }-P_{3}}{P_{1}^{\prime }-P_{1}}, \end{array}$$

(A20) $$\begin{array}{cc} W>0 & \Rightarrow \frac{\omega_{1}-\omega_{1}^{\prime }}{\omega_{2}-\omega_{2}^{\prime }}>\frac{P_{3}^{\prime }-P_{3}}{P_{1}^{\prime }-P_{1}}. \end{array}$$

Proceeding along the lines of Case (a) above, we again conclude that both gaps must shrink, with the lower gap bounded as follows:

(A21) $$\begin{array}{cc} \frac{T_{1}}{T_{2}}\omega_{1}^{\prime } & >\omega_{1}>\omega_{1}^{\prime }. \end{array}$$

Then, the conditions $Q_{1}>0$ and $Q_{2}<0$ combined with PWC can be expressed as:

(A22) $$\frac{\omega_{1}^{\prime }}{\omega_{2}^{\prime }}>\frac{\omega_{1}}{\omega_{2}}>\frac{\omega_{1}-\omega_{1}^{\prime }}{\omega_{2}-\omega_{2}^{\prime }}>\frac{P_{3}^{\prime }-P_{3}}{P_{1}^{\prime }-P_{1}}.$$

Hence, Cases (a) and (b) are consistent with both the energy gaps shrinking in the first quantum adiabatic process. Alternately, when we control the 3LS as per G-1, then both Cases (a) and (b) are permissible.

### Appendix A.4. When One Gap Is Kept Fixed

We determine the PWC when the lower energy gap remains fixed ($\omega_{1}=\omega_{1}^{\prime }$) during the Otto cycle. The expressions for work and heat are simplified to:

(A23) $$\begin{array}{cc} W & =\left(\omega_{2}-\omega_{2}^{\prime }\right)\left(P_{3}-P_{3}^{\prime }\right), \end{array}$$

(A24) $$\begin{array}{cc} Q_{1} & =\omega_{2}\left(P_{3}-P_{3}^{\prime }\right)+\omega_{1}\left(P_{1}^{\prime }-P_{1}\right), \end{array}$$

(A25) $$\begin{array}{cc} Q_{2} & =-\omega_{2}^{\prime }\left(P_{3}-P_{3}^{\prime }\right)-\omega_{1}^{\prime }\left(P_{1}^{\prime }-P_{1}\right). \end{array}$$

There are four possible relationships between the hot and cold distributions.

Case (a) yields

(A26) $$\frac{P_{3}}{P_{2}}>\frac{P_{3}^{\prime }}{P_{2}^{\prime }}\Rightarrow \frac{\omega_{2}}{T_{1}}<\frac{\omega_{2}^{\prime }}{T_{2}}.$$

Also, using the fixed lower gap condition, we obtain

(A27) $$\frac{P_{1}}{P_{2}}>\frac{P_{1}^{\prime }}{P_{2}^{\prime }}\Rightarrow \frac{1}{T_{1}}>\frac{1}{T_{2}}.$$

However, PWC from Equation (A23) requires $\omega_{2}>\omega_{2}^{\prime }$. Since we have assumed $T_{1}>T_{2}$, we find Equation (A27) in contradiction. Consequently, Case (a) is not permissible as an Otto engine when the lower gap is fixed and the upper gap shrinks.

Similarly, for Case (b), W>0 implies $\omega_{2}<\omega_{2}^{\prime }$, since $P_{3}<P_{3}^{\prime }$. Then, we have

(A28) $$\frac{P_{3}}{P_{2}}<\frac{P_{3}^{\prime }}{P_{2}^{\prime }}\Rightarrow \frac{\omega_{2}}{T_{1}}>\frac{\omega_{2}^{\prime }}{T_{2}},$$

and

(A29) $$\frac{P_{1}}{P_{2}}<\frac{P_{1}^{\prime }}{P_{2}^{\prime }}\Rightarrow \frac{1}{T_{1}}<\frac{1}{T_{2}}.$$

Since we require $\omega_{2}<\omega_{2}^{\prime }$, Equation (A28) cannot hold. Thus, Case (b) does not allow for the engine.

Now, consider Case (c), which yields

(A30) $$\frac{P_{3}}{P_{1}}>\frac{P_{3}^{\prime }}{P_{1}^{\prime }}\Rightarrow \frac{\omega }{T_{1}}<\frac{\omega^{\prime }}{T_{2}},$$

where $\omega (\omega^{\prime })$ represent the sum of the gaps under respective conditions. PWC from Equation (A23) requires $\omega_{2}>\omega_{2}^{\prime }$. Therefore, we must have $\omega >\omega^{\prime }$, as the other gap is fixed. Thus, Equation (A30) holds, as well as $Q_{1}>0,\;\;Q_{2}<0$ for Case (c). Finally, for Case (d), we obtain $Q_{1}<0,\;\;Q_{2}>0$. Thus, the engine is not a permissible operation with Case (d). Therefore, only the majorization condition permits its operation as an engine. Similarly, we can treat the case of the fixed upper gap ($\omega_{2}=\omega_{2}^{\prime }$) while the lower gap is shrinking.
